# Supplementary material for: WordCommentsAnalyzer: A windows software tool for qualitative research
Source: F1000Res. 2018 Sep 4;7:536. Originally published 2018 May 3. [Version 2] doi: 10.12688/f1000research.14819.2 (PMC6137411; doi:10.12688/f1000research.14819.2)
Supplement: Supplementary file 3 [file f1000research-7-17614-s0002.tgz › b2a79496-4ada-47c4-9682-a99ce867836d.zip!/Eight Iranian Health Journals/Archives of Rehabilitation_40.docx]

© Archives of Rehabilitation. This work is licensed under CC BY-NC 4.0 (https://creativecommons.org/licenses/by-nc/4.0/).

Volume & Issue: دوره 10 - شماره 4

Title: بررسی تأثیر تمرینات استقامتی-مقاومتی بر ظرفیت بدنی و شاخص‌های تن‌سنجی بیماران قلبی

http://rehabilitationj.uswr.ac.ir/article-1-416-fa.html

هدف: این تحقیق با هدف بررسی میزان تأثیر برنامه نوتوانی قلبی (تمرینات استقامتی و مقاومتی) بر عملکرد بدنی و نیز شاخص های چربی و قند خون بیماران قلبی صورت گرفت. روش بررسی: در این پژوهش نیمه تجربی و مداخله‌ای، ۲۰ بیمار قلبی پذیرش شده در بخش نوتوانی قلب بیمارستان شهید بهشتی همدان که در مرحله 2 نوتوانی بودند، به صورت ساده و در دسترس انتخاب و تمرینات مقاومتی و استقامتی به مدت ۲۴ جلسه (هفته‌ای ۳ جلسه) به آنها داده شد. ظرفیت بدنی، شاخص‌های تن سنجی و معیارهای پاراکلینیکی بیماران قبل و بعد از تمرینات ارزیابی و با آزمون آماری تی زوجی مورد مقایسه قرار گرفت. یافته‌ها: بعد از انجام تمرینات کاهش معناداری در میزان تری گلیسیرید (۰/۰۰۹=P)، کلسترول تام (۰/۰۱۹=P) و کلسترول تراکم بالا (۰/۰۳۶=P) نسبت به قبل از مداخله ملاحظه شد، در حالی که این کاهش در مورد قند خون (۰/۰۷۲=P) و کلسترول تراکم پایین (۰/۰۸۷=P) معنادار نبود. همچنین مسافت طی شده در مدت ۶ دقیقه توسط بیماران بعد از مداخله افزایش معناداری نسبت به قبل داشت (۰/۰۰۱>P) و میزان درک فشار تحمل شده توسط بیمار پس از انجام آزمون کاهش یافت (۰/۰۰۸=P). شاخص های چاقی از قبیل وزن (۰/۰۳۱=P) و ضخامت چربی زیر جلد (۰/۰۱۷=P) نیز پس از برنامه نوتوانی کاهش معناداری داشت. نتیجه‌گیری: برنامه نوتوانی قلبی شامل تمرینات استقامتی و مقاومتی موجب کاهش عوامل خطر ساز در بیماران چاق مبتلا به بیماری قلبی – عروقی و نیز افزایش ظرفیت فیزیکی بیمار می گردد

Title: مقایسه درک ترکیبات استعاری بین دانش‌آموزان آسیب دیده شنوایی با دانش‌آموزان شنوا در مقطع راهنمایی

http://rehabilitationj.uswr.ac.ir/article-1-417-fa.html

هدف: آسیب شنوایی شایع ترین نقص هنگام تولد است. بسیاری معتقدند افراد آسیب دیده شنوایی از مشکلات زبانی از جمله درک استعاره ها رنج می برند. هدف مطالعه حاضر مقایسه درک ترکیبات استعاری دانش آموزان شنوا با دانش آموزان آسیب دیده شنوایی شدید مقطع راهنمایی می باشد. روش بررسی: در این پژوهش توصیفی- تحلیلی و مقایسه‌ای 50 دانش آموز شنوا و 25 دانش آموز آسیب دیده شنوایی به روش نمونه گیری تصادفی ساده از میان مدارس عادی و مدارس استثنایی ویژه ناشنوایان مقطع راهنمایی انتخاب شدند. برای انجام آزمون، از دو تکلیف محقق ساخته استفاده و در ابتدا روایی محتوایی و سپس با استفاده از روش آزمون-بازآزمون، پایایی تکلیف درک واژگان غیر استعاری و تکلیف درک ترکیبات استعاری ارزیابی شد. سپس تکلیف درک واژگان غیر استعاری اجرا و چنانچه آزمودنی به بیش از 75 درصد کلمات پاسخ صحیح داده و بقیه معیارهای انتخاب نمونه را دارا بود، تکلیف درک ترکیبات استعاری اجرا می شد. داده ها با استفاده از آزمون های آماری تی مستقل و تحلیل واریانس مورد تجزیه و تحلیل قرار گرفت. یافته‌ها: بین نمره درک ترکیبات استعاری دانش آموزان شنوا و آسیب دیده شنوایی تفاوت معناداری وجود داشت (P<0/001). بیشترین تعداد پاسخهای غلط دانش آموزان آسیب دیده شنوایی از نوع لفظی بود. در هیچ یک از دو گروه، میانگین پاسخهای صحیح در سه پایه تحصیلی اختلاف معناداری نداشت. نتیجه‌گیری: دانش‌آموزان آسیب دیده شنوایی در درک ترکیبات استعاری در طول 3 پایه تحصیلی رشدی نداشته و پاسخ های غلط ایشان به آزمون، حاکی از فهم لفظی ایشان از ترکیبات استعاری است. تفاوت قابل ملاحظه دانش آموزان شنوا و آسیب دیده شنوایی در درک ترکیبات استعاری نشان دهنده نیاز ویژه آنها به آموزش در این زمینه می باشد.

Title: بررسی میزان تأثیر آسیب خفیف تا متوسط بینایی و شنوایی بر فعالیت‌های روزمره زندگی و تعادل سالمندان

http://rehabilitationj.uswr.ac.ir/article-1-418-fa.html

هدف: پژوهش حاضر با هدف بررسی میزان تأثیر آسیب خفیف تا متوسط دو حس شنوایی و بینایی ناشی از کهولت بر فعالیتهای روزمره زندگی و تعادل سالمندان انجام گرفت. روش بررسی: در پژوهش تحلیلی و مورد- شاهدی حاضر، از بین 370 نفر سالمند ساکن در 6 آسایشگاه شمال تهران، 36 نفر بر اساس معیارهای لحاظ شده به روش نمونه گیری ساده و در دسترس انتخاب و آزمونهای شنوایی و بینایی به صورت اودیومتری و جداول E (اسنلن)، بر روی نمونه ها انجام و براساس این آزمونها افراد در 4 گروه قرار گرفتند: ا- افرادی که فقط دارای مشکل بینایی بودند 2- افرادی که فقط دارای مشکل شنوایی بودند 3- افراد دارای هر دو مشکل شنوایی و بینایی 4- افرادی که مشکل شنوایی و بینایی نداشتند. سپس آزمون های مربوط به فعالیت روزمره زندگی در سالمند و تست تعادلی برگ برای افراد هر 4 گروه انجام شد. نتایج با استفاده از آزمونهای تحلیل واریانس، کروسکال والیس و آزمون تعقیبی بن‌فرونی مورد تجزیه و تحلیل واقع شد. یافته‌ها: خطر نسبی وابستگی در امور مربوط به فعالیتهای روزمره زندگی در ابتلا به اختلال بینایی خفیف تا متوسط 2/46 و خطر نسبی وابستگی در ابتلا به اختلال شنوایی خفیف تا متوسط برابر 1/5 بود. بیشترین خطر نسبی وابستگی وقتی بود که هر دو آسیب به طور توأم وجود داشت (خطر نسبی=2/85). آسیب شنوایی بر تعادل بی تأثیر و آسیب بینایی هر چند خفیف، بر تعادل تأثیرگذار بود (P<0/001). توأم شدن دو آسیب شنوایی و بینایی بر تعادل تأثیرگذار بود (P<0/001)، ولی باعث افزایش اختلال در تعادل نسبت به آسیب یک حس به تنهایی نشد. نتیجه‌گیری: کاهش شنوایی ناشی از کهولت حتی اگر در حد خفیف تا متوسط باشد، بر فعالیتهای روزمره زندگی تأثیرگذار است، اما این میزان کاهش شنوایی بر تعادل سالمندان تأثیرگذار نیست، مگر آن که با اختلال بینایی توأم باشد.

Title: مقایسه تصویر بدنی معلولین ورزشکار با معلولین و غیرمعلولین

http://rehabilitationj.uswr.ac.ir/article-1-419-fa.html

هدف: تحقیق حاضر با هدف مقایسه تصویر بدنی مردان معلول ورزشکار با مردان معلول و غیر معلول غیر ورزشکار انجام شد. روش بررسی: در این مطالعه مقطعی- مقایسه ای، سه گروه پنچاه نفره از معلولین ورزشکار، معلولین غیر ورزشکار و غیر معلول غیر ورزشکار مرد شهر تهران به طور تصادفی و خوشه ای به ترتیب از اماکن ورزشی مخصوص معلولین، آسایشگاه معلولین کهریزک و ادارات دولتی انتخاب و تصویر بدنی آنها مورد مقایسه قرار گرفت. ابزارهای به کار رفته شامل پرسشنامه اطلاعات فردی محقق ساخته و همچنین پرسشنامه استاندارد خود توصیفی بدنی بود که از 11 خرده مقیاس و کل بدن تشکیل شده است. روشهای آماری مورد استفاده شامل آزمون تحلیل واریانس یک طرفه و آزمون تعقیبی نیومن- کلز بود. یافته‌ها: تصویر بدنی معلولین ورزشکار در خرده مقیاسهای قدرت، استقامت، هماهنگی، انعطاف پذیری، عزت نفس، فعالیت بدنی، لیاقت ورزشی و کل بدن (P&ge0/001) به طور معنا داری از معلولین و غیر معلولین غیر ورزشکار بهتر بود. همچنین در خرده مقیاسهای ظاهر بدن (P=0/012)، سلامت عمومی (P=0/001) و چربی بدن (0/002=P)؛ معلولین ورزشکار به طور معنا داری بهتر از معلولین غیر ورزشکار بودند، اما با غیر معلولین غیر ورزشکار تفاوت معناداری نداشتند. نتیجه‌گیری: با توجه به اینکه افراد معلول ورزشکار به دلیل انجام فعالیت بدنی مستمر تصویر بدنی مطلوبتری نسبت به معلولین و غیر معلولین غیر ورزشکار دارند، لذا توصیه می شود که شرایط انجام فعالیتهای ورزشی برای معلولین مهیا گردد.

Title: مقایسه اولتراسونیک تغییرات ضخامت عضلات شکم در وضعیت‌های مختلف در مردان مبتلا به کمردرد مزمن غیراختصاصی و مردان سالم

http://rehabilitationj.uswr.ac.ir/article-1-420-fa.html

هدف: مقایسه تغییرضخامت عضلات شکم در وضعیتهای مختلف نشسته در مردان سالم و مردان مبتلا به کمردرد مزمن غیر اختصاصی با استفاده از سونوگرافی هدف این مطالعه می‌باشد. روش بررسی: در این مطالعه مورد–شاهدی 20 مرد مبتلا به کمردرد مزمن غیراختصاصی از مراجعین کلینیک‌های فیزیوتراپی دانشگاه علوم بهزیستی و توانبخشی و ‏20 مرد سالم به صورت ساده و در دسترس و با لحاظ کردن معیارهای ورود و خروج انتخاب شدند. ضخامت عضلات عرضی شکم، مایل داخلی، مایل خارجی و راست شکمی در سمت راست توسط سونوگرافی نوع B    (7.5 مگاهرتز خطی) در وضعیت‌های طاقباز (وضعیت1)، نشسته روی صندلی (وضعیت2)، نشسته روی توپ ژیمناستیک (وضعیت3) و نشسته روی توپ ژیمناستیک با بلند کردن پای چپ از روی زمین (وضعیت4) در انتهای دم و بازدم در دو گروه اندازه گیری شد. داده‌های حاصل با استفاده از آزمونهای تی زوجی و مستقل مورد مقایسه و تجزیه و تحلیل قرار گرفت. یافته‌ها: ضخامت عضلات عرضی شکم و مایل داخلی در هر 4 وضعیت و عضله راست شکمی فقط در وضعیت 4 در هر دو گروه سالم و بیمار در انتهای بازدم بیشتر از دم بود (۰/۰۰۱>P). تغییرات ضخامت عضلات عرضی شکم، مایل داخلی و راست شکمی در مقایسه وضعیتهای ۳ با ۲، ۴ با ۲ و ۴ با ۳ و عضله مایل خارجی فقط در مقایسه وضعیتهای ۴ با ۲ و ۴ با ۳ در هر دو گروه سالم و بیمار در دو حالت دم و بازدم معنادار شد  (۰/۰۰۱>P). ضخامت عضله عرضی شکم افراد سالم در وضعیت4 بیشتر از افراد بیمار (۰/۰۰۱>P) و ضخامت عضله راست شکمی افراد بیمار در وضعیت4 بیشتر از افراد سالم بود (۰/۰۱=P). نتیجه‌گیری: عضلات شکم به تغییرات پاسچر پاسخ می دهند و این عضلات با کاهش ثبات وضعیت نشسته به طور خودکار وارد عمل می شوند. در بیماران کمردردی فعالیت عضله عرضی شکم کاهش یافته و فعالیت عضله راست شکمی افزایش می یابد.

Title: مقایسه نیازهای توجهی کنترل راه‌رفتن در ورزشکاران سالم و مبتلا به بی‌ثباتی عملکردی مچ پا

http://rehabilitationj.uswr.ac.ir/article-1-421-fa.html

هدف: با توجه به میزان شیوع بالای بی ثباتی عملکردی مچ پا در میان ورزشکاران و نیاز این افراد برای انجام چند فعالیت به صورت همزمان در زمین مسابقه، این مطالعه با هدف مقایسه نیازهای توجهی کنترل راه رفتن بین ورزشکاران مبتلا به بی ثباتی عملکردی مچ پا و همتایان سالم انجام شد. روش بررسی: در این مطالعه مقطعی مورد- شاهدی، 16ورزشکار مبتلا به بی ثباتی عملکردی مچ پا و 16 همتای سالم با نمونه گیری ساده انتخاب شدند. این مطالعه از نوع سه عاملی مختلط و با الگوی تکلیف دو گانه بوده و از سیستم تحلیل حرکت استفاده شد. زمان و طول گام، زمان، طول، فرکانس و پهنای قدم و زمان ایستایش (Stance) و زمان نوسان (Swing) قدم در حالی که داوطلبین 2 دشواری از تکلیف راه رفتن (شامل راه رفتن روی دستگاه تردمیل با دو سرعت 2/1 و 3/6 کیلومتر در ساعت) و 2 تحمیل تکلیف شناختی (شامل تحمیل صفر و شمارش معکوس) را تجربه می کردند، اندازه گیری و داده های حاصل با استفاده از آزمون های آماری مجذور خی، تحلیل واریانس سه طرفه مختلط و تی مستقل تجزیه و تحلیل شد. یافته‌ها: آزمون تحلیل واریانس مختلط 2×2×2 (2 گروه×2 دشواری تکلیف راه رفتن ×2 دشواری تکلیف شناختی) اثر متقابل گروه در وضعیت را بر طول گام معنادار نشان داد (P=0/03)، به نحوی که در مواجهه با تکلیف شناختی در بیماران افزایش طول گام ولی در گروه سالم کاهش طول گام دیده شد. همچنین فرکانس قدم در گروه بیمار معنادار شد (P=0/049). نتیجه‌گیری: به نظر می رسد که بی ثباتی عملکردی مچ پا با افزایش نیازهای توجهی کنترل راه رفتن همراه است. بنابراین تحمیل تکلیف شناختی ممکن است بتواند به عنوان یک استراتژی مؤثر در برنامه ورزشی و درمانی مبتلایان به بی ثباتی عملکردی مچ پا منظور گردد.

Title: بررسی پایایی نسخه فارسی مقیاس سنجش شدت خستگی در افراد مبتلا به مولتیپل اسکلروزیس

http://rehabilitationj.uswr.ac.ir/article-1-422-fa.html

هدف: پژوهش حاضر با هدف فراهم ‌سازی ابزاری پایا به زبان فارسی جهت سنجش شدت خستگی در افراد مبتلا به مولتیپل اسکلروزیس از طریق ترجمه و معادل‌سازی پرسشنامه «مقیاس سنجش شدت خستگی» به زبان فارسی و بررسی پایایی نسخه فارسی معادل‌سازی شده در افراد مبتلا به مولتیپل اسکلروزیس انجام شد. روش بررسی: تحقیق حاضر یک مطالعه غیرتجربی از نوع متدولوژیک و اعتبارسنجی می‌باشد. پرسشنامه «مقیاس سنجش شدت خستگی» طی سه مرحله طبق روش بین المللی ارزیابی کیفیت زندگی به زبان فارسی ترجمه و معادل‌سازی شد و برای بررسی تکرارپذیری، در دو نوبت با فاصله حداکثر 3 هفته در اختیار 20 بیمار (15 زن و 5 مرد) فارسی زبان که طبق تشخیص پزشک مبتلا به مولتیپل اسکلروزیس بوده و از سال 1380 تا 1386 جهت دریافت خدمات پزشکی و توانبخشی به کلینیک سینا مراجعه کرده بودند قرار گرفت. روش نمونه گیری به صورت ساده بود. داده‌های به دست آمده از لحاظ تکرارپذیری در دفعات آزمون و همخوانی درونی آیتم‌ها، از طریق محاسبه ضریب آلفای کرونباخ، ضریب همبستگی پیرسون و همبستگی درون طبقه‌ای تحت تجزیه و تحلیل آماری قرار گرفت. یافته‌ها: همخوانی درونی آیتم‌های پرسشنامه « مقیاس سنجش شدت خستگی» با محاسبه ضریب آلفای کرونباخ برابر 0/96 بود که نشان می‌دهد آیتم‌های مقیاس فوق یک مفهوم را ارزیابی می‌کنند. آزمون ضریب همبستگی درون طبقه‌ای (ICC) که برای بررسی تکرارپذیری نسبی نسخه فارسی پرسشنامه «مقیاس سنجش شدت خستگی» در دفعات آزمون به کار رفته بود محاسبه و برابر 0/93 به دست آمد که تکرارپذیری خوب نسخه فارسی پرسشنامه «مقیاس سنجش شدت خستگی» را نشان می‌دهد. نتیجه‌گیری: پرسشنامه « مقیاس سنجش شدت خستگی» از تکرارپذیری بالایی برخوردار است و می‌توان با استفاده از آن میزان شدت خستگی و تأثیر درمان‌های مختلف را روی افراد مبتلا به مولتیپل اسکلروزیس بررسی نمود.

Title: بررسی ویژگی‌های گفتار و زبان کودکان حاصل از درمان‌های کمک‌باروری

http://rehabilitationj.uswr.ac.ir/article-1-423-fa.html

هدف:‌ در این مطالعه به بررسی برخی ویژگی های گفتار و زبان کودکان حاصل از درمانهای کمک باروری پرداخته شده است. روش بررسی: مطالعه از نوع توصیفی – تحلیلی و نمونه گیری به صورت ساده از جامعه در دسترس و به شکل تمام شمار بوده است. 50 کودک صفر تا ۴۹ ماهه از طریق مصاحبه با والدین و مشاهده بالینی کودک توسط آسیب شناس گفتار و زبان مورد ارزیابی قرار گرفته و اطلاعات اخذ شده در پرسشنامه‌ای ثبت شد. طلاعات این پرسشنامه بر اساس مقیاس نشانگان زبان ابتدایی دو (ELM–2)، آزمون میانگین طول گفته (MLU) و بسته مهارت‌های دهانی اطفال (POSP) می باشد. برای تجزیه و تحلیل داده‌ها از آزمونهای تی مستقل، کولموگروف اسمیرنوف، من ویتنی یو و مجذور خی استفاده شد. یافته‌ها: از 50 کودک مورد بررسی 33 نفر (%66) حاصل از درمانهای تزریق داخل سیتوپلاسمی اسپرم (ICSI) و بقیه حاصل از تلقیح (تزریق اسپرم) داخل رحمی (IUI) و لقاح آزمایشگاهی (IVF) بودند. میانگین طول گفته بر حسب واژه در آزمودنی های 19 تا ۴۹ ماهه 3/3 واژه بود. %8 این کودکان در ساختمان اندامهای گویایی و %6 در عملکرد اندامهای گویایی اختلال نشان دادند. یافته های این پژوهش نشان داد در میانگین طول گفته بر حسب واژه (۰/۰۶۹=P)، سن شروع صداسازی بازتابی (۰/۱۲۵=P)، تعداد واژه های با معنی (۰/۰۷۹=P) و ساختمان و عملکرد اندام‌های گویایی (۰/۳۲۷=P) بین دو جنس تفاوت معناداری وجود ندارد. در صورتی که در سن شروع قان و قون آهنگین تفاوت بین دو جنس معنا دار بود (0/026=P). نتیجه‌گیری:‌ گفتار و زبان کودکان حاصل از درمانهای کمک باروری در محدوده طبیعی قرار دارد. برای تعمیم یافته ها نیاز به مطالعات جامع تری می باشد.

Title: بررسی پیوستگی 7 جایگاه ژنی برای ناشنوایی غیرسندرومی آتوزومی مغلوب در خانواده‌های ایرانی

http://rehabilitationj.uswr.ac.ir/article-1-425-fa.html

هدف: نقص شنوایی فراوان ترین بیماری حسی– عصبی می‌باشد که به دو فرم سندرومی و غیرسندرومی مشاهده می شود. هدف این مطالعه، بررسی پیوستگی ۷ جایگاه ژنی مسئول در ناشنوایی غیر سندرومی آتوزومی مغلوب در خانواده‌های ایرانی می باشد. روش بررسی: در این مطالعه توصیفی، 41 خانواده دارای حداقل ۲ فرد مبتلا به ناشنوایی غیرسندرومی آتوزومی مغلوب که جهش در جایگاههای ژنی D‏FNB1, DFNB2, DFNB3, DFNB4, DFNB6 ,DFNB7/11 ,DFNB8/10 DFNB9, DFNB12, DFNB16, DFNB18, DFNB21, DFNB23, DFNB29, و DFNB4 در آنها منفی بود، به صورت ساده و در دسترس انتخاب و 7 جایگاه ژنی D‏FNB67 ,DFNB37 ,DFNB36 ,DFNB31 DFNB30 ,DFNB28 و DFNB22 با ژنهای مشخص در آنها بررسی شد. نقشه یابی هوموزیگوسیتی در مورد این خانواده ها با استفاده از مارکرهای چند شکل (STR) انجام شد. یافته‌ها: 3 خانواده در 3 جایگاه ژنی DFNB28 ,DFNB30 و DFNB31 به طور مشخص پیوستگی نشان دادند. نتیجه‌گیری: در این تحقیق تقریباً %7 از علل ناشنوایی غیرسندرومی آتوزومی مغلوب در بین جمعیت ایران مشخص شد.
